# SMARCB1/INI1 loss in skull base conventional chordomas: a clinicopathological and molecular analysis

**DOI:** 10.3389/fonc.2023.1160764

**Published:** 2023-06-30

**Authors:** Alberto Righi, Stefania Cocchi, Margherita Maioli, Matteo Zoli, Federica Guaraldi, Elisa Carretta, Giovanna Magagnoli, Ernesto Pasquini, Sofia Melotti, Gianfranco Vornetti, Caterina Tonon, Diego Mazzatenta, Sofia Asioli

**Affiliations:** ^1^ IRCCS Istituto Ortopedico Rizzoli, Bologna, Italy; ^2^ IRCCS Istituto delle Scienze Neurologiche di Bologna, Bologna, Italy; ^3^ Department of Biomedical and Neuromotor Sciences (DIBINEM), University of Bologna, Bologna, Italy

**Keywords:** skull base, chordoma, prognosis, SMARCB1/INI1, FISH analysis

## Abstract

**Introduction:**

The loss of SMARCB1/INI1 protein has been recently described in poorly differentiated chordoma, an aggressive and rare disease variant typically arising from the skull base.

**Methods:**

Retrospective study aimed at 1) examining the differential immunohistochemical expression of SMARCB1/INI1 in conventional skull base chordomas, including the chondroid subtype; 2) evaluating SMARCB1 gene deletions/copy number gain; and 3) analyzing the association of SMARCB1/INI1 expression with clinicopathological parameters and patient survival.

**Results:**

65 patients (35 men and 30 women) affected by conventional skull base chordoma, 15 with chondroid subtype, followed for >48 months after surgery were collected. Median age at surgery was 50 years old (range 9-79). Mean tumor size was 3.6 cm (range 2-9.5). At immunohistochemical evaluation, a partial loss of SMARCB1/INI1 (>10% of neoplastic examined cells) was observed in 21 (32.3%) cases; the remaining 43 showed a strong nuclear expression. Fluorescence *in situ* hybridization (FISH) analysis was performed in 15/21 (71.4%) cases of the chordomas with partial SMARCB1/INI1 loss of expression. Heterozygous deletion of SMARCB1 was identified in 9/15 (60%) cases and was associated to copy number gain in one case; no deletion was found in the other 6 (40%) cases, 3 of which presenting with a copy number gain. No correlations were found between partial loss of SMARCB1/INI1 and the clinicopathological parameters evaluated (i.e., age, tumor size, gender, tumor size and histotype). Overall 5-year survival and 5-year disease-free rates were 82% and 59%, respectively. According to log-rank test analysis the various clinico-pathological parameters and SMARCB1/INI1 expression did not impact on overall and disease free-survival.

**Discussion:**

Partial loss of SMARCB1/INI1, secondary to heterozygous deletion and/or copy number gain of SMARCB1, is not peculiar of aggressive forms, but can be identified by immunohistochemistry in a significant portion of conventional skull base chordomas, including the chondroid subtype. The variable protein expression does not appear to correlate with clinicopathological parameters, nor survival outcomes, but still, it could have therapeutic implications.

## Introduction

Skull base chordomas represent a heterogeneous group of tumors, including different histotypes (i.e., conventional, chondroid, poorly differentiated and dedifferentiated types) with different clinical behavior (1–5).

SWI/SNF-related matrix-associated actin-dependent regulator of chromatin subfamily B member 1 (SMARCB1), also known as integrase interactor 1 (INI1), is a critical component of a chromatin-remodeling protein complex (4, 6). Recent studies have described the immunohistochemical loss of SMARCB1/INI1 protein in poorly differentiated chordoma associated with *SMARCB1* gene deletions at fluorescence *in situ* hybridization (FISH) examination, mainly deriving from large, homozygous deletions at 22q11 locus (4, 6–8). The loss of SMARCB1/INI1 protein could potentially serve as theoretical basis for evaluating the efficacy of new targeted therapies, i.e., Enhancer of Zeste homologue 2 (EZH2) inhibitors (Tazemetostat), histone deacetylase inhibitors, and CDK4 inhibitors (4, 7, 9–11).

Some studies have recently suggested the partial loss of SMARCB1/INI1 expression at immunohistochemistry as a poor prognostic marker of outcome, being associated with higher recurrence rates and shorter survival in patients with other tumor types, including colorectal, pancreatic, uterine and sinonasal carcinomas (12–16).

Genetic studies have demonstrated that conventional chordomas are characterized by very low to modest mutation burden, and are mainly characterized by large copy number loss, typically involving chromosomes 1p, 3, 9q, 10, 13, and 14, and a small number of copy number gains on chromosome 7 and 1q (4, 9). Loss of chromosome 22 and/or heterozygous deletion of *SMARCB1* seems to be a rare event in conventional chordomas, although data are referred to small series (4, 17–19).

This study aimed at evaluating the differential immunohistochemical expression of SMARCB1/INI1 in conventional skull base chordoma, including chondroid subtype, and the presence of *SMARCB1* gene deletion/copy number gain by FISH. Potential associations of SMARCB1/INI1 expression with different clinicopathological parameters and survival outcomes were then analyzed.

## Materials and methods

### Patient selection

Patients with conventional - including chondroid variant - and with poorly differentiated chordoma (1, 2), naïve for surgery and radiation therapy, operated via endoscopic endonasal approach from 1998 to 2017 in a tertiary care center (Programma Neurochirurgia Ipofisi - Pituitary Unit, IRCCS Istituto delle Scienze Neurologiche di Bologna, Italy), followed by Radiation-therapy, and with a clinico-radiological follow-up ≥48 months. Formalin-fixed paraffin-embedded (FFPE) tumor tissue of adequate size and quality was required to perform morphologic, immunohistochemical and molecular evaluations. The pathologist selected the most representative tumor fragments for size and quality (i.e., maximum representation of neoplastic cells and lowest portions of extra-chordoma tissues and necrosis). All the original tumor slides were reviewed, and the diagnosis was confirmed independently by two pathologists (SA and AR) with a confirmation of immunohistochemical expression of brachyury and pan-cytokeratin AE1/AE3. Three cases of poorly differentiated chordomas diagnosed at the Programma Neurochirurgia Ipofisi-Pituitary Unit, IRCCS Istituto delle Scienze Neurologiche di Bologna, Italy, for which FFPE tissue was available, were also included. Ethical committee approval was obtained from the Comitato Etico di Area Vasta Emilia Centro on 01/04/2019 (protocol # CE-AVEC: 184/2019/OSS/AUSLBO).

#### Immunohistochemical analysis

The tissue was fixed in 4% buffered formalin, processed and embedded in paraffin; 4μm-thick tissue sections were then cut and heated at 58°C for 2 h. Immunohistochemical staining was performed using an automated immunostainer following the manufacturer’s guidelines (Ventana BenchMark -Ventana Medical Systems, Tucson AZ, USA) using an antibody anti-INI-1 (MRQ-27; Cell Marque), a mouse monoclonal antibody ready to use at the concentration of 0,4 µg/ml (MRQ-27; Cell Marque). Antibody detection was performed using UltraView DAB Detection Kit (Ventana Medical Systems, Tucson AZ, USA). Immunohistochemical evaluation was performed as previously described (20). The percentage of cells stained was determined evaluating all neoplastic areas in the whole of the obtained slides in each case, independently assessed by two pathologists (AR, SA). This evaluation was done visually and a comparison between immunohistochemical expression of SMARCB1/INI1 and the signals of FISH analysis was done. Immunohistochemical staining grades were defined as intact (strong nuclear staining in malignant cells), deficient (completely unstained nuclei in malignant cells), and reduced (very weak but still noticeable nuclear staining in malignant cells), using the strong staining of normal background cells as reference (20, 21). Strong homogeneous nuclear staining in the background (including inflammatory cells, stromal fibroblasts, vascular endothelial cells, and/or normal epithelial cells) served as an internal control and was considered a prerequisite for immunohistochemical interpretation. Only unequivocal staining of the nuclei in viable tumor tissue (necrotic areas were excluded) was analyzed. The evaluations were performed on 200X of magnification, evaluating the mean of SMARCB1/INI1 loss, when present, for each mm^2^.

#### FISH analysis

FISH was performed to assess *SMARCB1* gene deletion using a commercial SPEC SMARCB1/22q12 Dual colour Probe (ZytoVision, Bremerhaven, Germany), according to manufacturer’s instructions. The probe included a 545 kb sequence mapping in 22q11.23 region (ZyGreen fluorochrome labeled) harboring *SMARCB1* gene, and a 335 kb sequence mapping in 22q12.1-q12.2 region (ZyOrange fluorochrome labelled) harboring *KREMEN1* gene, used as internal control probe, to help in detecting chromosome 22q large deletions. As previously described (22), FISH was performed on interphase nuclei using the Histology FISH accessory kit (Dako, Glostrup, Denmark), according to the manufacturers’ protocol. Briefly, 3 μm-thick FFPE tissue sections were mounted on positively charged slides. Slides were heated overnight at 60°C, deparaffinized with xylene, and dehydrated with ethanol. Samples and probes were co-denaturated in a Dako Hybridizer (Dako, Glostrup, Denmark) at 75°C for 10 minutes and incubated overnight at 37°C. Slides were then washed in stringent solution for 10 minutes at 63°C and stained with DAPI (Vector Laboratories, Inc. Burlingame CA, USA). Signal analysis was performed in combination with SMARCB1/INI1 nuclear expression correlation. For each slide, a minimum of 100 nuclei within the marked tumor area with intact morphology were scored using an Olympus BX41 fluorescent microscope (Tokyo, Japan) at 100X of magnification. Nuclei with no signal and signals in overlapped nuclei were considered non-informative and were not analyzed to avoid truncation or overlapping artifact. The presence of two copies of the *SMARCB1* gene with a 1:1 ratio with the control probe was considered as the normal copy number pattern. A heterozygous co-deletion pattern (or large deletion) was defined if one allele copy of both *SMARCB1* gene and control probe were lost, with a ratio of 1:1. Copy number gain was defined as the presence of extra copies of both *SMARCB1* and control probe. A Color View III CCD camera soft imaging system (Olympus) was used to capture images, then analyzed with a CytoVision imaging software version 7.5 (Leica Biosystem Richmond Inc, USA).

#### Statistical analysis

Disease-free Survival (DFS) was defined as the time between treatment completion and first disease relapse. Patients free from disease were censored at last follow up. Overall Survival (OS) was defined as the time between treatment completion and death or last follow-up. Descriptive statistic was used to report patient and clinical characteristics. T-test or Wilcoxon Mann-Whiney test were used to analyze continuous variables; chi-squared test or Fisher’s exact test to analyze categorical variables. Kolmogorov-Smirnov and Shapiro-Wilk test were used to verify normal distribution of continuous variables. Time-to event measures were estimated using Kaplan–Meier method, and log-rank test was used to compare different parameters. All p-values were two-sided and a p<0.05 was considered as statistically significant. All statistical analyses were performed using SAS software 9.4 (SAS Institute Inc., Cary, NC).

## Results

Sixty-five patients with conventional skull base chordoma, including 35 (53.8%) men and 30 (46.2%) women, with a median age at first surgery of 50 years old (range 9-79), were enrolled. Mean tumor size at presentation was 3.6 cm (range 2-9.5 cm) (see **Table 1**). Histologically, 50 (76.9%) were conventional chordomas, while 15 (23.1%) were chordomas of chondroid subtype, characterized by extracellular matrix mimicking hyaline cartilage inside physalifourous neoplastic cell proliferation in the majority of the neoplastic evaluated areas (1, 2). The mean of Ki-67 labeling index was 4% (range 1-25).

**Table 1:** Main clinicopathological characteristics and distribution according to SMARCB1/INI1 immunohistochemical expression.

Parameters	All samples (n=65)	SMARCB1/INI1 + (n=44)	SMARCB1/INI +/- (n=21)	P value
**Age** (median, range; years)	50 (9-79)	51.6 (17-79)	49.2 (9-73)	0.5956
Age (N, %)				
≤ 50 years	34 (52.3)	23 (52.3)	11 (52.4)	0.9935
>50 years	31 (47.7)	21 (47.7)	10 (47.6)	
Gender (N, %)				
Male	35 (53.8)	21 (47.7)	14 (66.7)	0.1520
Female	30 (46.2)	23 (52.3)	7 (33.3)	
Tumor size (N, %)				
< 3cm	10 (15.4)	5 (11.4)	5 (23.8)	0.2714
≥ 3cm	55 (84.6)	39 (88.6)	16 (76.2)	
Histological subtype (N, %)				
conventional	50 (76.9)	34 (77.3)	16 (76.2)	1.000
chondroid	15 (23.1)	10 (22.7)	5 (23.8)	
Ki-67 (N, %)				
≤ 3%	36 (55.4)	24 (54.6)	12 (57.1)	0.8438
**>**3%	29 (44.6)	20 (45.4)	9 (42.9)	

At immunohistochemical evaluation, a partial loss of SMARCB1/INI1 (between 10% and 40% of neoplastic cells evaluated) was observed in 21 (32.3%) cases; the remaining 44 (67.7%) cases showed a strong nuclear expression in all neoplastic cells (see **Supplementary File Table 1**). None of conventional/chondroid chordoma cases displayed complete loss of SMARCB1/INI1 loss. Poorly differentiated chordomas presented loss of SMARCB1/INI1 in all evaluated neoplastic cells.

**Figure 1:**
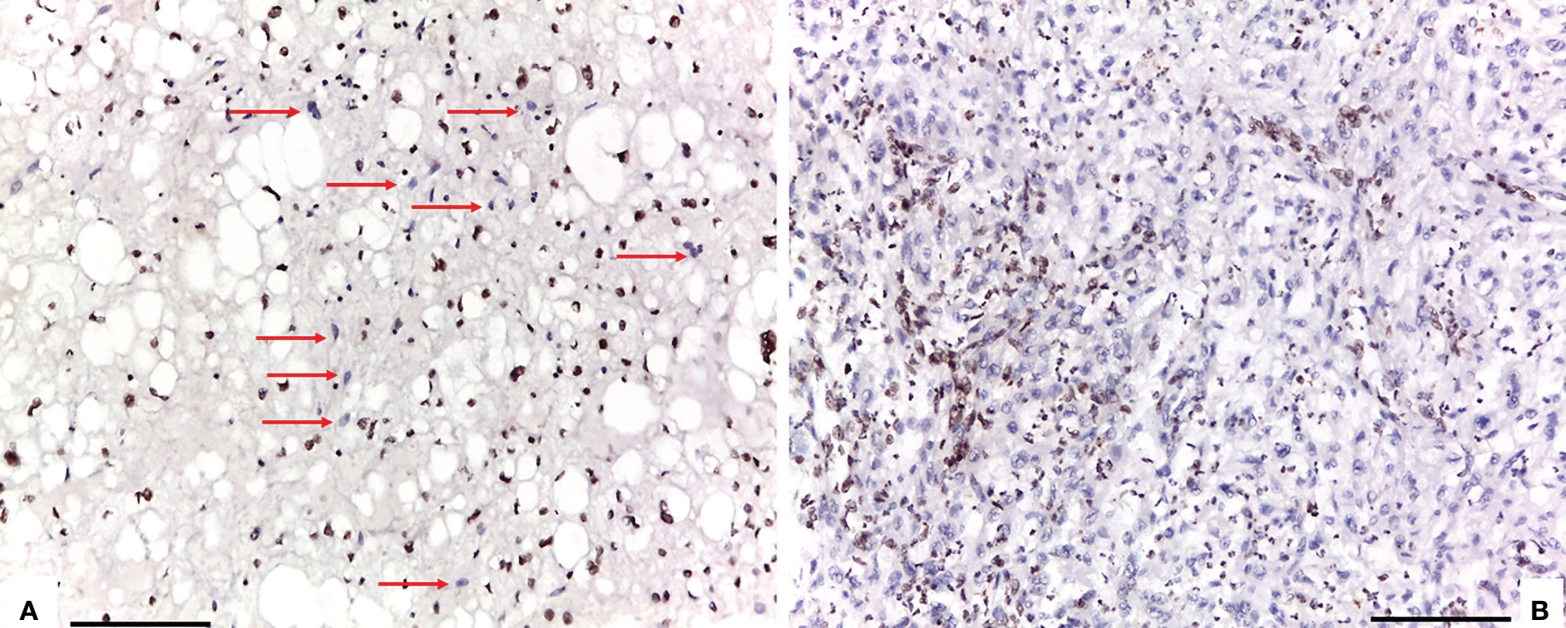
Two different staining patterns in neoplastic areas of focal loss of SMARCB1/INI1: **(A)** an example of a case that showed a mosaic pattern of protein loss, with isolated single/small foci of negative cells (red arrows) closed to other foci of cells that retained SMARCB1/INI1; **(B)** an example of a case that showed protein loss in large areas, looking like ‘subclonal’ foci within the tumor (A, B: 100X of magnification, Scale bar=75 μm).

**Figure 2:**
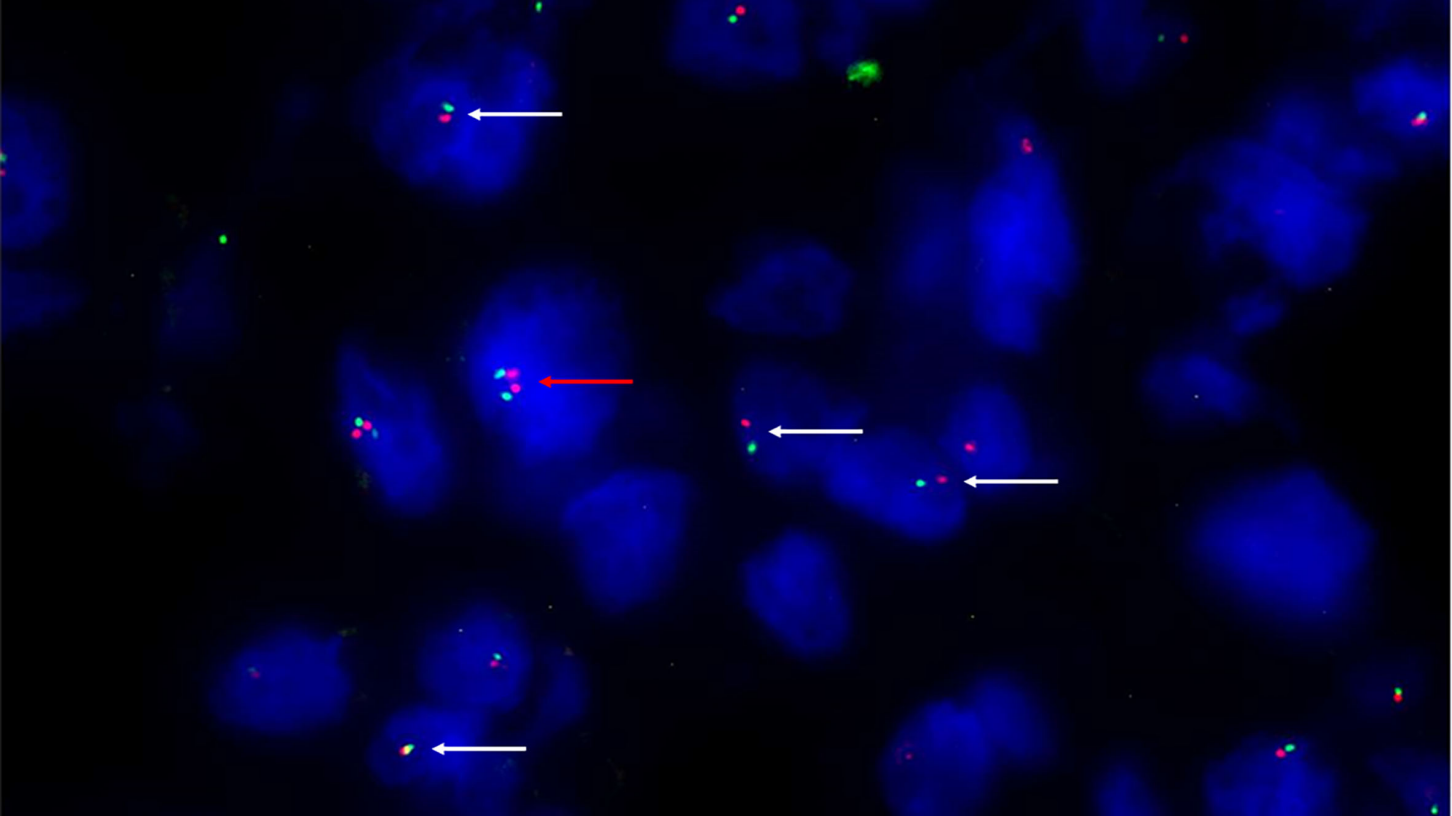
Fluorescence *in situ* hybridization (FISH) with SPEC SMARCB1/22q12 Dual Color Probe detected, in a representative tumoral area. Monoallelic co-deletion pattern: only one copy of SMARCB1/INI1 (green signal) and one copy of 22q12 (red signal) were observed in most tumor cells (white arrows). A cell without deletion is shown in the field as internal control (red arrow). (200X of magnification).

Conventional chordomas with focal loss of SMARCB1/INI1 displayed two different staining patterns in neoplastic areas: 13 cases showed a mosaic pattern of protein loss, with isolated single/small foci of negative cells closed to other foci of cells that retained SMARCB1/INI1 (**Figure 1A**); 8 cases showed protein loss in large areas, looking like ‘subclonal’ foci within the tumor (**Figure 1B**). No differences in clinicopathological factors between the two different staining patterns were observed. Regardless to the pattern of SMARCB1/INI1 expression, no association could be established between SMARCB1/INI1 expression and gender, age, tumor size, Ki67 and histological subtype (see **Table 1**). FISH analysis could be performed with a readable signal in 15/21 (71.4%) cases of conventional chordomas with a partial immunohistochemical loss of SMARCB1/INI1 expression. Six cases did not show hybridized signal due to poor tissue quality, and were thus considered inadequate for FISH scoring. FISH analysis demonstrated the presence of heterozygous deletion of *SMARCB1* in 9/15 (60%) cases in over 10% of tumors cells (range 10% to 80%, **Figure 2**), and was associated with a copy number gain in one case. No deletion was observed in the other 6 (40%) cases, 3 of which presenting with a copy number gain of *SMARCB1* (**Figure 2**). FISH identified homozygous *SMARCB1* deletions in all 3 cases of poorly differentiated chordoma.

**Figure 3:**
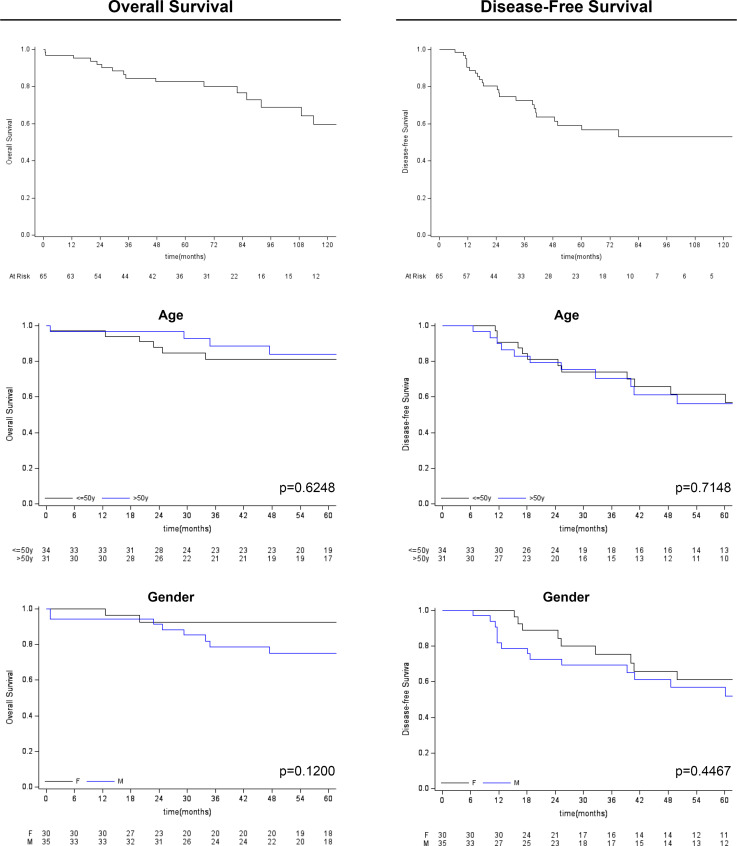
Kaplan-Meier survival analysis (overall survival and disease-free survival) for age and gender variables.

**Figure 4:**
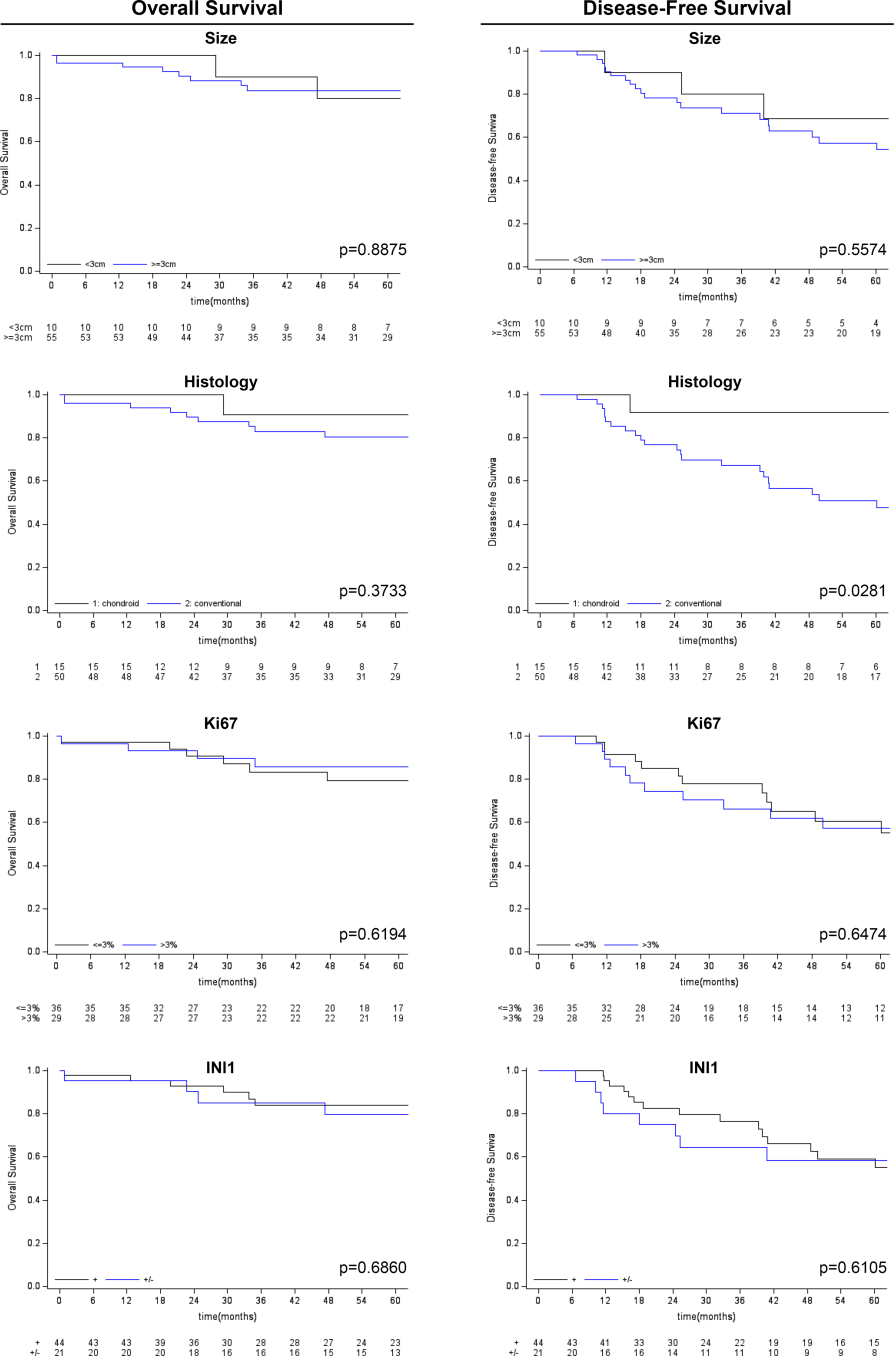
Kaplan-Meier survival analysis (overall survival and disease-free survival) for other clinico-pathological variables considered (tumor size, histological subtype, Ki-67 and SMARCB1/INI1 immunohistochemical expression).

Follow-up duration after treatment completion was 80 months (range, 51-127). Overall 5-year survival and 5-year disease-free rates were 83% (95%CI: 69.9-90.5) and 59% (95%CI:44.5-71.3), respectively. Univariate analysis showed that the risk of recurrence/metastases was higher for conventional than chondroid chordoma (p=0.0281). Among all considered parameters, only the histological subtype impacted on DFS, while no predictor of OS was identified (see **Tables 2**, **3**; **Figures 3**, **4**).

**Table 2:** Results from univariate Kaplan-Meier models for OS and DFS.

	5 years-OS % (95%CI)	p-value	5 years-DFS % (95%CI)	p-value
**Entire sample**	82.6 (69.9-90.5)		59.2 (44.5-71.3)	
Age (N, %)				
≤50 years	81.3 (62.8-91.1)	0.6248	61.8 (41.1-77.0)	0.7148
>50 years	84.1 (62.7-93.8)		56.1 (34.1-73.3)	
Gender (N, %)				
Male	75.3 (56.4-86.8)	0.1200	56.8 (36.8-72.6)	0.4467
Female	92.7 (73.7-98.1)		61.2 (38.2-77.8)	
Tumor size (N, %)				
< 3cm	80.0 (40.9-94.6)	0.8875	68.6 (30.5-88.7)	0.5574
≥ 3cm	83.6 (69.7-91.5)		57.4 (41.1-70.7)	
Histological subtype (N, %)				
conventional	80.5 (65.7-89.4)	0.3733	50.8 (34.7-64.9)	0.0281
chondroid	90.9 (50.8-98.7)		91.7 (53.9-98.8)	
Ki-67 (N, %)				
≤ 3%	79.4 (59.4-90.3)	0.6194	60.4 (39.1-76.3)	0.6474
**>**3%	85.6 (66.0-94.4)		57.4 (36.0-73.9)	
SMARCB1/INI1 immunohistochemical expression (N, %)				
positive	84.0 (67.5-92.5)	0.6860	59.1 (40.3-73.8)	0.6105
negative	79.9 (54.8-92.0)		58.4 (33.6-76.8)	

**Table 3:** Results from univariate Kaplan-Meier models for OS and DFS according the different pattern of partial loss of SMARCB1/INI1 by immunohistochemistry.

	5 years-OS % (95%CI)	p-value overall	p-value subclonal vs mosaic
**Pattern**		0.7246	0.5671
Subclonal	75.0 (31.5-93.1)		
Mosaic	83.3 (48.2-95.6)		
Positive	84.0 (67.5-92.5)		
	**5 years-DFS % (95%CI)**		
**Pattern**		0.1859	0.1785
Subclonal	42.9 (9.8-73.4)		
Mosaic	67.7 (34.9-86.5)		
Positive	59.1 (40.3-73.8)		

## Discussion

Skull base chordomas represent a heterogeneous group of neoplasia, extremely difficult to be eradicated by surgical and adjuvant means, although typically slow-growing. New therapeutic targeted therapies are currently under investigation, including EZH2 inhibitors (Tazemetostat) (4, 10, 11, 23). EZH2 is a catalytic subunit of the histone methyltransferase PCR2 polycomb repressive complex whose overexpression promotes oncogenesis (24). Agents targeting EZH2 have shown to induce tumor regression and promote radiation sensitivity in models of SMARCB1/INI1-deficient tumors, including poorly differentiated chordomas, malignant rhabdoid tumors and epithelioid sarcomas (4, 10, 11). Differently from most of atypical teratoid/rhabdoid tumors, in chordomas, loss of SMARCB1/INI1 expression at immunohistochemistry results from a homozygous deletion of the SMARCB1 gene (4, 9, 25), and has been reported not only in poorly differentiated variants (in which it represents a diagnostic hallmark) (4, 6, 9, 26, 27), but also in a case of conventional chordoma with transformation to poorly differentiated chordoma (17), and in another case of conventional chordoma with dedifferentiated sarcomatous components (28).

Based on literature data, total loss of SMARCB1/INI1 immunohistochemical expression associated with the presence of a homozygous deletion of the *SMARCB1* gene is correlated with aggressive clinical behavior of chordomas (8, 17, 27–29). Only few series have evaluated SMARCB1/INI1 immunohistochemical expression in association with FISH analysis in conventional chordoma. Overall, the study by Mobley et al. (30) and by Hassellbatt et al. (4) found the retention of SMARCB1/INI1 without a recurrent deletion of *SMARCB1* region in 14 out of 24 cases. Conversely, Yadav et al. (27) described 2 cases of conventional chordomas with loss of immunohistochemical expression of SMARCB1/INI1 associated with loss of *SMARCB1* locus, and Wen reported a single case of extra-axial conventional chordoma with a partial loss of SMARCB1/INI1 at immunohistochemistry despite no deletion of *SMARCB1* detected by FISH analysis (9). Therefore, to best of our knowledge, this is the largest study aimed at investigation the incidence of SMARCB1/INI1 loss in of conventional skull base chordomas, accounting for >95% of chordomas (1, 2, 8). Immunohistochemical analysis demonstrated a partial loss of SMARCB1/INI1 in 10 to 40% of neoplastic cells in 21/65 (32.3%) cases, and no correlations between partial SMARCB1/INI1 loss and clinicopathological parameters. Furthermore, unlike poorly differentiated chordoma and other types of carcinomas (8, 12, 13, 21, 31), log-rank test analysis showed no impact of SMARCB1/INI1 expression on overall and disease free-survival.

From a molecular point of view, SMARCB1/INI1 loss of function may be caused by gene deletions, inactivating mutations, or epigenetic modifications. *SMARCB1* heterozygosity is considered the main underlying mechanism and can be revealed by FISH analysis with very high sensitivity (7, 9, 32). FISH analysis on FFPE tissue in SMARCB1-deficient tumors has been proven to be a reliable test to investigate large homozygous or heterozygous deletions at 22q11.12 (33). Due to cross-hybridizations of chromosome 22 alpha satellites to other centromeric regions, probes specific for 22q12.1-q12.2 region are frequently used as control for chromosome 22 copy number detection. However, since the SMARCB1 gene and the control probe used are only 5.5 Mb away (chromosome bands 22q11.23 and 22q12.1-q12.2, respectively), secondary regional deletions may occur in SMARCB1-deleted tumors. Many studies have stated that in SMARCB1-deficient tumors large deletions covering also the EWSR1 gene locus (chromosome band 22q12.2) can occur (7, 30, 33–35), so demonstrating the deletion of a large portion of the long arm of chromosome 22. More advanced genomics and epigenetics sequencing approaches should be used in future in depth studies of chromatin modifier SMARCB1/INI1. In our series, 21 cases of conventional chordoma displayed partial loss of SMARCB1/INI1 expression at immunohistochemistry; 6 (28.6%) showed no readable signal at FISH analysis, due to the poor tissue quality. Of the remaining 15, 9 showed heterozygous large 22q deletion encompassing the entire *SMARCB1* gene locus, while 6 cases had no deletion, confirming a previous observation (9). Consistent with previous studies (4, 9), homozygous *SMARCB1* loss was observed in the 3 cases of poorly differentiated, but not in conventional chordoma. Interestingly, one case with *SMARCB1* heterozygous deletion showed copy number gain of *SMARCB1*; control probe suggested a gain in the 22q region. This pattern was observed also in 3 cases without *SMARCB1* deletion. Anyway, since the 12 patients with heterozygous 22q deletion and/or a copy number gain of *SMARCB1* showed reduced expression of SMARCB1/INI1 at immunohistochemistry, it is possible that this partial loss of SMARCB1/INI1 is a marker of the accumulation of additional mutations as suggested by Bai et al., who reported complex copy number alterations, including also the deletion of 22q, without apparent recurrent oncogene mutations in conventional chordoma (29). Therefore, EZH2 inhibitors (Tazemetostat) may prove to be beneficial in treating conventional chordoma, as recently demonstrated *in vitro* and *in vivo* studies and in patient-derived xenograft model (23, 36). Although promising, these data are preliminary and collected retrospectively, thus need to be confirmed by larger prospective studies, possibly multicentric because of the rarity of the disease.

In conclusion, the partial loss of SMARCB1/INI1, secondary to heterozygous deletion and/or copy number gain of SMARCB1, can be identified by immunohistochemistry in a significant portion of conventional chordomas, and is not peculiar of aggressive cases. The different protein expression does not appear to correlate with clinicopathological parameters, nor survival outcomes. However, it could still have therapeutic implications.

## Author’s note

This work was partially presented as platform presentation at the 112th Meeting of the United States and Canadian Academy of Pathology in New Orleans, Louisiana, USA, 11-16 March, 2023.

## Data availability statement

The original contributions presented in the study are included in the article/**Supplementary Material**. Further inquiries can be directed to the corresponding author.

## Ethics statement

The studies involving human participants were reviewed and approved by Comitato Etico di Area Vasta Emilia Centro on 01/04/2019 (protocol # CE-AVEC: 184/2019/OSS/AUSLBO). Written informed consent to participate in this study was provided by the participants’ legal guardian/next of kin.

## Author contributions

All authors contributed to the study conception and design. Material preparation, data collection and analysis were performed by AR, SC, MM, MZ, GM, EP, SM, GV, CT, DM, and SA. EC performed statistical analysis. The draft of the manuscript was written by AR, FG, SC, and SA. All authors read and approved the final manuscript.
